# Neurofilament Light Chain and Intermediate HTT Alleles as Combined Biomarkers in Italian ALS Patients

**DOI:** 10.3389/fnins.2021.695049

**Published:** 2021-09-03

**Authors:** Assunta Ingannato, Silvia Bagnoli, Salvatore Mazzeo, Valentina Bessi, Sabrina Matà, Monica Del Mastio, Gemma Lombardi, Camilla Ferrari, Sandro Sorbi, Benedetta Nacmias

**Affiliations:** ^1^NEUROFARBA Department, University of Florence, Florence, Italy; ^2^SOD Neurologia 1, Dipartimento Neuromuscolo-Scheletrico e Degli Organi di Senso, Azienda Ospedaliero Universitaria Careggi, Florence, Italy; ^3^IRCCS Fondazione Don Carlo Gnocchi, Florence, Italy

**Keywords:** amyotrophic lateral sclerosis, neurofilament light chain, CAG repeat expansion, HTT gene, biomarkers

## Abstract

**Objective:**

To study the possible implication of the two biomarkers, intermediate alleles (IAs) of the Huntingtin (HTT) gene and neurofilament light chain (NfL) levels in plasma, in amyotrophic lateral sclerosis (ALS) patients.

**Methods:**

We analyzed IAs in a cohort of 106 Italian ALS patients and measured the plasma NfL levels in 20% of the patients of the cohort. We correlated the two biomarkers with clinical phenotypes.

**Results:**

Intermediate alleles were present in 7.5% of the patients of our cohort, a frequency higher than that reported in general population. Plasma NfL levels increased with age at onset (*p* < 0.05). Patients with bulbar onset (BO) had higher plasma NfL concentration (CI −0.61 to −0.06, *p* = 0.02) and a later age at onset of the disease (CI −24.78 to −4.93, *p* = 0.006) with respect to the spinal onset (SO) form. Additionally, two of the patients, with IAs and plasma NfL concentration lower with respect to normal alleles’ carriers, presented an age at onset higher than the mean of the entire cohort.

**Conclusion:**

According to our findings, plasma NfL and IAs of HTT gene may represent potential biomarkers in ALS, providing evidence of a possible implication in clinical phenotype.

## Introduction

Amyotrophic lateral sclerosis (ALS) is a neurodegenerative disorder characterized by degeneration of upper and lower motoneurons, leading to progressive weakness, paralysis, and, in the end, death typically within 3–5 years from symptom onset (Hardiman et al., 2011; van Es et al., 2017). To date, the causes of ALS remain unknown; most cases are sporadic, whereas only 5–10% of the patients have a familiar form caused by a mutation in a known gene (Taylor et al., 2016). The most common pathogenic mutations are in the causative genes superoxide dismutase 1 (SOD1), TAR DNA binding protein (TARDBP or *TDP-43*), and chromosome 9 open reading frame 72 (C9orf72) (Yousefian-Jazi et al., 2020). The diagnosis of ALS is based on clinical findings and arrives only many months after symptom onset (Paganoni et al., 2014), so there is an urgency to find biological markers that could be helpful in diagnosis and prognosis and that could be included in common medical practice. Neurofilament light chain (NfLs) is the most promising biomarker in neurodegenerative diseases, and in the last years, it has been studied extensively in different neurological diseases (Bacioglu et al., 2016; Khalil et al., 2018; Gaetani et al., 2019). NfLs are subunits of neurofilaments, neuron-specific proteins belonging to the intermediate filament family, highly expressed in large caliber myelinated axons (Herrmann and Aebi, 2016). NfL levels increase in biological fluids, such as cerebrospinal fluids (CSF) and blood, proportionally to the degree of the axonal damage (Reiber, 1994). An elevated concentration of NfLs in CSF or blood indicates neuronal degeneration (Gaiottino et al., 2013). An innovative ultrasensitive single-molecule technology, called Simoa, can detect proteins at femtomolar concentration in blood, allowing precise quantification of NfLs (Rissin et al., 2010). Recently, several studies have focused on the potential diagnostic performance of NfLs as a biomarker in ALS (Lu et al., 2015; Li et al., 2018; Poesen and Van Damme, 2018; Verde et al., 2019). Their levels in CSF and blood are higher in ALS patients compared with healthy controls and also correlate with the disease progression rate and survival (Tortelli et al., 2012; Boylan et al., 2013; Lu et al., 2015; De Schaepdryver et al., 2018; Gille et al., 2019). Furthermore, recent studies are also trying to highlight the role of CAG repeat expansion in different neurological disorders (Dewan et al., 2021; Leotti et al., 2021). Expansions of the CAG repeat in the ATXN2 gene, which cause spinocerebellar ataxia type 2, have been associated with increased risk of ALS (Sproviero et al., 2017), while patients carrying CAG triplet expansion in the Huntingtin (HTT) gene in a range between 27 and 35, referred to as an intermediate allele (IA), showed motor and cognitive changes (Cubo et al., 2016; Jot, 2019; Savitt and Jankovic, 2019). A correlation between susceptibility to neurodegenerative diseases and HTT CAG repeat expansion was reported in 2019, suggesting that IAs might have a role also in the pathogenesis of Alzheimer’s disease, increasing disease risk (Menéndez-González et al., 2019). The reported frequency of IAs in the general population is around 6% (Savitt and Jankovic, 2019), not so different from that observed in neurodegenerative disorders (Menéndez-González et al., 2019). For these reasons, further studies are still needed. The aim of our study was to test, for the first time, in an Italian cohort of ALS patients, the implication in the disease of the two biomarkers, plasma NfLs and IAs of the HTT gene focusing on disease susceptibility, age at onset, and site of onset (bulbar versus spinal). Finally, we examined whether there was a correlation between the two biomarkers.

## Materials and Methods

### ALS Patients and Clinical Characteristics

The study does include ALS patients, with a defined diagnosis according to El Escorial diagnostic criteria for ALS (Brooks, 1994), recruited at the Neurological Clinic I of Careggi Hospital in Florence and consecutively enrolled from March 2009 to November 2020. Patients carrying a pathogenic mutation in a causative gene (SOD1, TDP43, and C9orf72) were excluded from the study. In fact, pathogenic mutation in causative genes could potentially act as a confounding factor in the overall analysis. Moreover, the presence of all other disease processes was an exclusion criterion. The study finally included a cohort of 106 Italian ALS patients where, at the first visit, all patients underwent a neurologic and functional assessment and venipuncture for blood collection. A minority of the patients (seven patients, with concomitant dementia at the onset) were evaluated with an extensive neuropsychological battery as described in more detail elsewhere (Bracco et al., 1990) and with SAND for language evaluation (Screening for Aphasia in NeuroDegeneration) (Catricalà et al., 2017), and received a clinical diagnosis of FTD according to the current criteria, including the behavioral variant (bv-FTD) and the non-fluent variant of primary progressive aphasia (nfv-PPA) (Neary et al., 1998; Gorno-Tempini et al., 2011). The study protocol was approved by the local ethics committee and conducted in accordance with the provisions of the Declaration of Helsinki.

### Genetic Testing

High-molecular-weight DNA was isolated from whole blood using a QIAamp DNA blood mini QIAcube Kit (Qiagen, Germany), as described by the manufacturer. The amount of DNA for each sample has been determined using a NanoDrop ND-3300^®^ Fluorospectrometer. DNA samples were aliquoted and stored at −20°C until use. HTT CAG repeat expansion was determined by a polymerase chain reaction (PCR) amplification assay using the following primers: 5′-[6-FAM] GACCCTGGAAAAGCTGATGA-3′ and 5′-GGCTGAGGAAGCTGAGGAG-3′. The forwarded primer was modified with 6-carboxyfluorescein (6-FAM), a fluorescent dye for labeling oligonucleotides (Jama et al., 2013). The size of the PCR product was determined by capillary electrophoresis using an ABI 3130X automated DNA sequencer and the GeneMapper version 4.0 software (Applied Biosystems). A set of HTT CAG alleles, whose lengths were confirmed by DNA sequencing, was used to provide standard size. CAG repeat expansions were considered as follows: normal alleles with CAG expansion under 27 repeats, IAs with 27–35 repetitions, and pathologic allele with expansions size > 35 repeats.

### Plasma Sample Collection and NfL Analysis

Plasma was isolated from peripheral blood sample within 2 h of collection. Blood sample was centrifuged at 1300 rcf at 4° for 10 min, and the supernatant was immediately frozen and stored at −80°C until tested. Plasma NfL concentration was detected with the ultrasensitive single-molecule array (Simoa) technology provided by Quanterix Corporation (Lexington, MA, United States) (Rissin et al., 2010), on the automatized Simoa SR-X platform (GBIO, Hangzhou, China), following the instructions of the manufacturer. A Simoa NF-Light SR-X kit (Cat. No 103400) for human samples was used according to the protocol provided by Quanterix. All plasma samples were analyzed in a single run basis. Plasma samples and controls were diluted at a 1:4 ratio and measured in duplicate with calibrators. A calibration curve was calculated from measurements of serially diluted calibrators. The lower limit of quantification (LLOQ) and the limit of detection (LOD) provided by the kit were 0.316 and 0.0552 pg/ml, respectively. The quality control with low NfL concentration had a mean concentration of 5.08 pg/ml; the quality control with high NfL concentration had a mean of 169 pg/ml.

### Statistical Analysis

Statistical analysis was performed using R software v4.0.3 (The R Foundation) and SPSS software version 27 (IBM SPSS Statistics). We tested the correlations between continuous variables using Pearson’s correlation analysis; *p* < 0.05 was set as significant. Multiple linear regression was performed between the log function of NfL measurement and clinical parameters. Shapiro–Wilk’s test was executed to test the data normal distribution. To evaluate variable differences between groups, we used independent-samples *t*-test and Mann–Whitney *U*-test. Welch *t*-test was run when the assumption of homogeneity of variances was violated. To test whether the difference between two proportions is statistically significant, we used Fisher’s exact test.

## Results

### Italian ALS Cohort: Clinical Phenotype

The Italian cohort included 106 ALS patients (Table 1); 51 were female (48.1%) and 55 were male (51.9%). Disease clinical presentation at onset was ALS for 99 patients (93.4%) and ALS and bv-FTD for six patients (5.7%), and one patient (0.9%) showed ALS and nfv-PPA. The age at onset was available for 94 patients of the entire cohort, with a mean age of 67.04 ± 11.54 years. About 66% (70 out of 106) of ALS patients had a spinal onset (SO); 34% (36 patients) had ALS with a bulbar onset (BO). The mean age at onset of SO was 63.66 ± 11.91 years (64 out of 70 patients); the mean age at onset of BO was 74.30 ± 6.232 years (30 out of 36 patients).

**TABLE 1:** Clinical information of the Italian cohort of ALS patients.

	*ALS patients n = 106*
***Gender***	
*Female, n (%)*	51 (48.1)
*Male, n (%)*	55 (51.9)
***Site of onset***	
*SO, n (%)*	70 (66)
*BO, n (%)*	36 (34)
***Age at onset*** *(n = 94), mean*	67.04 ± 11.54
***Clinical presentation at onset***	
*ALS, n (%)*	99 (93.4)
*ALS and bv-FTD, n (%)*	6 (5.7)
*ALS and nfv-PPA, n (%)*	1 (0.9)

*Abbreviations: ALS, amyotrophic lateral sclerosis; SO, spinal onset; BO, bulbar onset; bv-FTD, behavioral variant of frontotemporal dementia; nfv-PPA, non-fluent variant of primary progressive aphasia.*

### IAs in ALS Patients

Out of 106 ALS, eight patients (7.5%) were carrying IAs of the HTT gene, and 98 patients (92.5%) presented normal alleles. No one showed a pathological allele expansion. Seven patients had IAs and SO, and one patient had IA and BO. Of the 98 ALS patients with normal alleles, 63 had SO and 35 had BO. There was no statistically significant association between IAs’ presence and site at onset, as assessed by Fisher’s exact test (*p* = 0.174), and neither with gender (*p* = 0.316). Moreover, IAs’ presence was not linearly related with age at onset [*F*(1,92) = 0.41, *p* = 0.840, and adjusted *R*^2^ = −0.010]. A Mann–Whitney *U*-test was run, and the mean ranks of age at onset for patients with IAs (41.94) and normal alleles (48.02) were not statistically significantly different, *U* = 299.5, *z* = −0.604, *p* = 0.546, using an exact sampling distribution for *U* (Dineen and Blakesley, 1973). The mean age at onset of IA carriers (eight out of 94) was 66.25 ± 7.78 years, and in normal allele carriers (86 out of 94), it was 67.12 ± 11.86 years.

### Plasma NfL Levels in ALS Patients

The plasma sample was available for a subgroup of 21 patients out of 106 ALS patients. To gain normally distributed data for all independent variables, we used the log function of NfL concentration (LogNfL) for analysis. The mean of plasma LogNfL detected in the entire cohort was 1.98 ± 0.32 pg/ml. There was a statistically significant, moderate positive correlation between LogNfL and age at onset (β 0.039; *p* < 0.05). In our subgroup, the mean age at onset was 67.20 ± 14.617 years. A linear regression model established that there were significant linear relationships between LogNfL and clinical data, age at onset [*F*(1,18) = 4.96, *p* < 0.05, and adjusted *R*^2^ = 0.17], and site of onset [*F*(1,19) = 6.47, *p* < 0.05, and adjusted *R*^2^ = 0.21]. Moreover, a statistically significant linear relationship emerged between age at onset and site at onset [*F*(1,18) = 5.92, *p* < 0.05, and adjusted *R*^2^ = 0.21]. There was univariate normality, as assessed by Shapiro–Wilk’s test (*p* > 0.05). No significant relationship was found with gender [*F*(1,19) = 0.4, *p* = 0.535, and adjusted *R*^2^ = −0.031].

**FIGURE 1:**
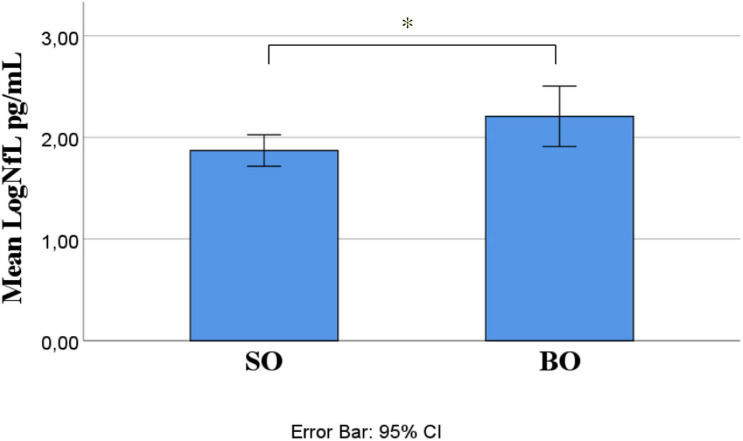
ALS site of onset compared for mean LogNfL levels. Mean LogNfL was higher in BO than SO (2.21 + 0.32 versus 1.87 + 0.27 pg/mL; *p* = 0.020). Abbreviations: LogNfL, log of NfL concentration; SO, spinal onset; BO, bulbar onset.

**FIGURE 2:**
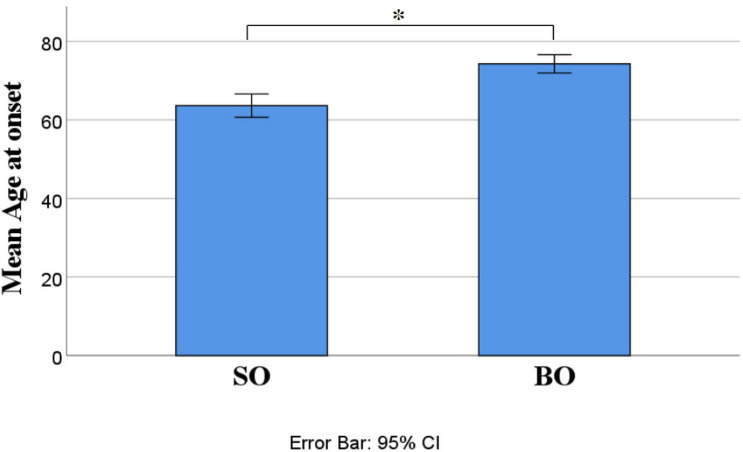
ALS site of onset compared for mean age at onset. Mean age at onset was higher in BO than SO (62 + 4.33 versus 76.86 + 1.74. *p* = 0.006). Abbreviations: LogNfL, log of NfL concentration; SO, spinal onset; BO, bulbar onset.

Out of 21 ALS patients, 14 (66.7%) had an SO and seven (33.3%) had a BO. Independent-samples *t*-test determined that LogNfL was higher in BO (2.21 ± 0.32 pg/mL) than SO (1.87 ± 0.27 pg/mL), a statistically significant difference of −0.34 (95% CI, −0.61 to −0.06), *t*(19) = −2.54, *p* = 0.020, and there was homogeneity of variances, as assessed by Levene’s test for equality of variances (*p* = 0.906) (Figure 1). We ran a receiver operating characteristic (ROC) analysis, and a cutoff level of 2.1028 pg/ml discriminated between BO and SO with 78.6% sensitivity and 71.4% specificity (95% CI 54.5–98.6%). The mean age at onset of SO was 62 ± 4.33 years, and BO had a mean age at onset of 76.86 ± 1.74 years, a statistically significant difference of −14.86 (95% CI, −24.78 to −4.93), *t*(15.377) = −3.18, *p* = 0.006 (Figure 2). No statistically significant differences in LogNfL emerged in gender.

Out of 21 patients, seven had a BO and normal alleles. Of 14 patients with SO, 12 had normal alleles and two were carrying IAs. The ALS age at onset of these two patients with IAs was, respectively, 73 and 77 years old.

### IAs and NfL in ALS Patients

No significant relationship was found between IA presence and LogNfL [*F*(1,19) = 1.07, *p* = 0.314 and adjusted *R*^2^ = 0.004]. Of 21 patients, two (9.52%) were carrying IAs with a mean LogNfL of 1.76 ± 0.2 pg/ml. The remaining 19 patients (90.47%) had normal alleles and a mean LogNfL of 2.0 ± 0.8 pg/ml.

## Discussion and Conclusion

The first aim of this study was to investigate for the first time in an Italian cohort of ALS patients the distribution of two potential biomarkers, IAs of the HTT gene and plasma NfL levels, and to examine their possible implication with clinical-demographic data, as gender, age at onset, and site of symptoms onset (SO or BO). Another aim of our work was to detect the possible interaction between the two biomarkers. IAs were present in 7.5% of our cohort, a frequency higher than that reported in the general population (Savitt and Jankovic, 2019), but with no one statistically significant association with clinical-demographic variables. Analysis of plasma NfL concentration in a subgroup (20%) of the cohort provided evidence for a statistically significant correlation with disease age at onset and site of onset. Plasma NfL levels increased with progressing age at onset. BO had higher plasma NfL concentration, suggesting a neurodegeneration degree more elevated than in spinal form. A significant correlation also resulted between the ALS site of onset and age at onset. Patients with BO had a later age at onset of disease. BO and higher age at symptom onset have been identified as negative prognostic factors for the disease (Arora and Khan, 2021; Ferraro et al., 2021), so we could hypothesize that elevated NfL concentration is a negative factor for the progression of the disease.

NfLs are highly expressed in axons (Lee and Cleveland, 1996). Damage to the axon scaffold, with a consequent impaired trafficking, has been supposed at the base of the ALS pathogenesis (Falzone et al., 2021). Several studies explored the potential value of NfL as a biomarker in ALS and demonstrated that ALS patients presented higher levels of NfL compared with healthy controls and with pathological controls affected by other forms of motor neuron disease (MND) (Steinacker et al., 2016; Xu et al., 2016; Rossi et al., 2018; Gagliardi et al., 2019). In our study, NfL concentration was analyzed with the Simoa platform in plasma samples, and we detected elevated NfL levels in Italian ALS patients. The mean concentration detected in our Italian cohort was comparable with the plasma data of ALS worldwide populations (Li et al., 2018; Gille et al., 2019; Verde et al., 2019). So, our study could contribute to extend the results of previous studies on NfL levels in ALS worldwide population and, also, prove the diagnostic value of plasma NfL in Italian ALS population as a non-invasive biomarker. Several studies investigated the NfL biomarker in Italian ALS patients but always in CSF (Gagliardi et al., 2019, 2021; Abu-Rumeileh et al., 2020). NfL levels are more elevated and, consequentially, easily detectable in CSF compared to peripheral blood, but a lumbar puncture is required. The innovative Simoa technology remarkably improved the analytical sensitivity, allowing measurement of the lowest NfL concentrations in blood samples (Rissin et al., 2010), and NfL levels in CFS and blood values are comparable (Kuhle et al., 2016; Steinacker et al., 2016). Blood-based biomarkers are preferable because they require minimally invasive collection compared to CSF sampling and also present the other important advantages to be simple, inexpensive, and readily available. In fact, clinical application of plasma NfLs, because they are an easily accessible biomarker, has been recently investigated in several neurodegenerative disorders, such as in multiple sclerosis, Alzheimer’s disease, frontotemporal dementia, Huntington’s disease, Parkinson and atypical Parkinsonian disorders, and traumatic brain injury (Shahim et al., 2016; Hansson et al., 2017; Ljungqvist et al., 2017; Barro et al., 2018; Lewczuk et al., 2018; Sánchez-Valle et al., 2018; Rojas et al., 2021; Sampedro et al., 2021). The potential of NfLs has also been indagated in oncology, microbiology, and infection diseases (Duffy, 2012; Schubert et al., 2015; Song et al., 2015).

We also observed that patients with incremented NfL levels were carrying normal alleles of the HTT gene. Two patients showed IAs and lower plasma NfL concentration. In addition, they had SO and an age at onset higher than the mean of the entire cohort. These preliminary data could indicate for IAs of the HTT gene a possible neuronal protective effect from neurodegeneration.

With regard to the second biomarker, the misfolded HTT protein, generated by the expansion of the CAG repeats in exon I of the HTT gene, is cleaved in mutated protein fragments that generate nuclear aggregates (Vonsattel and DiFiglia, 1998). Contrasting results were reported about their toxicity. A neuroprotective effect was seen in a HD transgenic mouse model with a strong reduction in susceptibility to excitotoxicity. It was suggested that at the basis of the imbalance toward the toxic or neuroprotective effect, there is the length of the fragments generated after the cleavage of the poly-Q stretch that could interact with proteins mediating resistance. Also, a full-length HTT protein folded differently than a shorter structure and could expose the exon I in a different manner leading to altered interactions (Slow et al., 2005; Zuchner and Brundin, 2008). Lee et al. (2018) described an “inverted U relationship” between the number of the CAG repeats of the HTT gene and a beneficial effect on cognitive functions. They demonstrated that the number of CAG repeats under 35 gives advantageous changes in brain structure and cognitive functions that becomes a disadvantage with an increasing length above 39 repetitions (Lee et al., 2017, 2018). Above the 39 CAG repeats, poly-Q tract would be non-functional in protein interactions, but below this threshold, HTT protein could show an increasingly greater flexibility, with an advantage in protein conformation and function and mediating changes in brain structure (Cattaneo et al., 2005; Schaefer et al., 2012; Caron et al., 2013). All these data support a possible neuronal protective effect of IAs in ALS patients.

Moreover, misfolded HTT protein and damaged axonal neurofilaments result in impaired trafficking and, consequently, in the loss of the neuronal connectivity. The impaired trafficking is a potential common mechanism at the base of ALS and HD pathogenesis (Morfini et al., 2013; Gatto et al., 2015).

In interpreting our findings, a few limitations should be considered. The sample size was relatively small, especially the subgroup (20% of patients) with data on plasma NfL. Moreover, not all patients were evaluated with a neuropsychological battery, so we did not control for the presence of associated cognitive symptoms that could act as confounding factors. Another limitation was the lack of a control group. IA frequency and plasma NfL levels were compared to literature data. This is a monocentric study, and a multicentric study would be useful to confirm these results. On the other hand, all samples were collected prospectively, processed, and stored using the same standardized method, and measurements of plasma NfL were done in a single batch, ensuring good reproducibility. As blood was collected at the first visit, when patients underwent a neurologic and functional assessment and venipuncture for blood collection, the correlation with disease progression was not considered. Our findings seem to reinforce the hypothesis that IAs could confer an advantage in degenerative brain disease, delaying the development of pathology and protecting from neuronal death. These preliminary findings indicate that both plasma NfL and IAs of the HTT gene may represent potential biomarkers for age at onset and site of onset (bulbar versus spinal), thus suggesting possible implication in clinical phenotype.

## Data Availability Statement

The raw data supporting the conclusions of this article will be made available by the authors, without undue reservation.

## Ethics Statement

The studies involving human participants were reviewed and approved by the AOU-Careggi Ethical Committee. The patients/participants provided their written informed consent to participate in this study.

## Author Contributions

BN and SS: project design. SMaz, VB, SMat, MD, GL, CF, and SS: recruitment of patients. AI and SB: genetics analysis. AI, SB, SMaz, and BN: acquisition and analysis of data. AI, SB, and BN: writing of the manuscript. AI, SB, VB, SMaz, and BN: revision of the manuscript. All authors contributed to the article and approved the submitted version.

## Conflict of Interest

The authors declare that the research was conducted in the absence of any commercial or financial relationships that could be construed as a potential conflict of interest.

## Publisher’s Note

All claims expressed in this article are solely those of the authors and do not necessarily represent those of their affiliated organizations, or those of the publisher, the editors and the reviewers. Any product that may be evaluated in this article, or claim that may be made by its manufacturer, is not guaranteed or endorsed by the publisher.
